# The genome and proteome of the *Kluyvera *bacteriophage Kvp1 – another member of the T7-like *Autographivirinae*

**DOI:** 10.1186/1743-422X-5-122

**Published:** 2008-10-20

**Authors:** Erika J Lingohr, Andre Villegas, Yi-Min She, Pieter-Jan Ceyssens, Andrew M Kropinski

**Affiliations:** 1 Public Health Agency of Canada, Laboratory for foodborne Zoonoses, Guelph, ON N1G 3W4, Canada; 2 Department of Chemistry, Queen's University, Kingston, Ontario K7L 3N6, Canada; 3 Laboratory of Gene Technology, Katholieke Universiteit Leuven, Kasteelpark Arenberg 21, Leuven, B-3001, Belgium; 4 Department of Molecular and Cellular Biology, University of Guelph, Guelph, Ontario, N1G 2W1, Canada

## Abstract

**Background:**

*Kluyvera*, a genus within the family *Enterobacteriaceae*, is an infrequent cause of human infections. Bacteriophage Kvp1, the only bacteriophage isolated for one of its species, *Kluyvera cryocrescens*, is a member of the viral family *Podoviridae*.

**Results:**

The genome of Kvp1, the first *Kluyvera cryocrescens*-specific bacteriophage, was sequenced using pyrosequencing (454 technology) at the McGill University and Genome Québec Innovation Centre. The two contigs were closed using PCR and the sequence of the terminal repeats completed by primer walking off the phage DNA. The phage structural proteome was investigated by SDS-PAGE and mass spectrometry.

**Conclusion:**

At 39,472 bp, the annotated genome revealed a closer relationship to coliphage T3 than T7 with Kvp1 containing homologs to T3 early proteins S-adenosyl-L-methionine hydrolase (0.3) and protein kinase (0.7). The quantitative nature of the relationships between Kvp1 and the other members of the T7-like virus genus (T7, T3, φA1122, φYeO3-12, Berlin, K1F, VP4 and gh-1) was confirmed using CoreGenes.

## Background

The T7-like bacterial viruses are members of the *Podoviridae *– phages with short tails – and are characterized by a simple but elegant temporal transcriptional control system [1]. The early genes are transcribed by the host RNA polymerase while the middle and late regions are transcribed by a single subunit phage-encoded RNA polymerase which recognizes unique 23 bp promoters sequences [2]. These viruses are one of the most common types of bacteriophages with 26–29 defined or tentative species according to the VIII report of the International Committee on the Taxonomy of Viruses [3,4]. Most of the host species are members of the γ-Proteobacteria (*Erwinia, Escherichia, Klebsiella, Morganella, Pseudomonas, Salmonella, Vibrio*, and *Yersinia*) but viral isolates also infecting α-Proteobacteria (*Caulobacter*, and *Rhizobium*) have been isolated. Fifteen T7-like phages have been sequenced and deposited with GenBank. As a result of a reanalysis, at the protein level, of relationships within the "T7-like viruses" this group of bacteriophages have been classified into the subfamily *Autographivirinae *which currently possesses three genera: T7-like, Sp6-like and φKMV-like viruses [5]. Kvp1, the first *Kluyvera cryocrescens*-specific bacteriophage, was isolated from the Matanza River in Buenos Aires (Argentina) by Gadaleta and Zorzopulos [6]. Morphologically this phage is a member of the *Podoviridae*. Eleven clones derived from *Alu*I or *Hae*III digestion of the viral DNA were sequenced, by these authors, revealing strong sequence similarity to coliphage T7. To further analyze the correct taxonomic position of this virus we have completed the sequence of its genome noting its very close similarity to *Yersinia *phage Berlin and coliphage T3.

## Results and discussion

Pyrosequencing (454 technology) has been used to determine the sequence of the genomes of *Bacillus thuringiensis *phage 0305φ8-36 [7] and coliphage JK98 [8], and, in this incidence, the genome of Kvp1. Sequencing resulted in 2 contigs with 53-fold coverage. While this type of sequencing can result in potential errors at oligonucleotide runs, none were observed in the data on Kvp1. The gap, representing 0 bp, was closed by PCR amplification and ABI sequencing; while the nature of the termini were verified by primer walking off phage DNA template. The total genome is 39,472 bp with 194 bp terminal direct repeats, and a base composition of 48.6 mol%G+C – characteristics remarkably consistent with other T7-like phages. By comparison, the genomes of T7-like phages range from 37.4 kb (*Pseudomonas *phage gh-1) to 45.4 kb (*Erwinia *phage Era103) while the reported terminal repeats range from *Yersinia *phage φA1122 at 148 bp to *Pseudomonas aeruginosa *phage LKD16 at 428 bp.

No tRNA genes were discovered, which was not an unexpected observation since no T7-like phages have been found to harbour them; 46 ORFs were delineated encoding protein products which show the strongest sequence similarity to gene products (Gps) from *Yersinia *phage Berlin (NC_008694). To investigate the relationships further we employed two homology tools, one of which function at the DNA sequence level (Mauve) and one, CoreGenes, which compares proteins.

**Figure 1:**
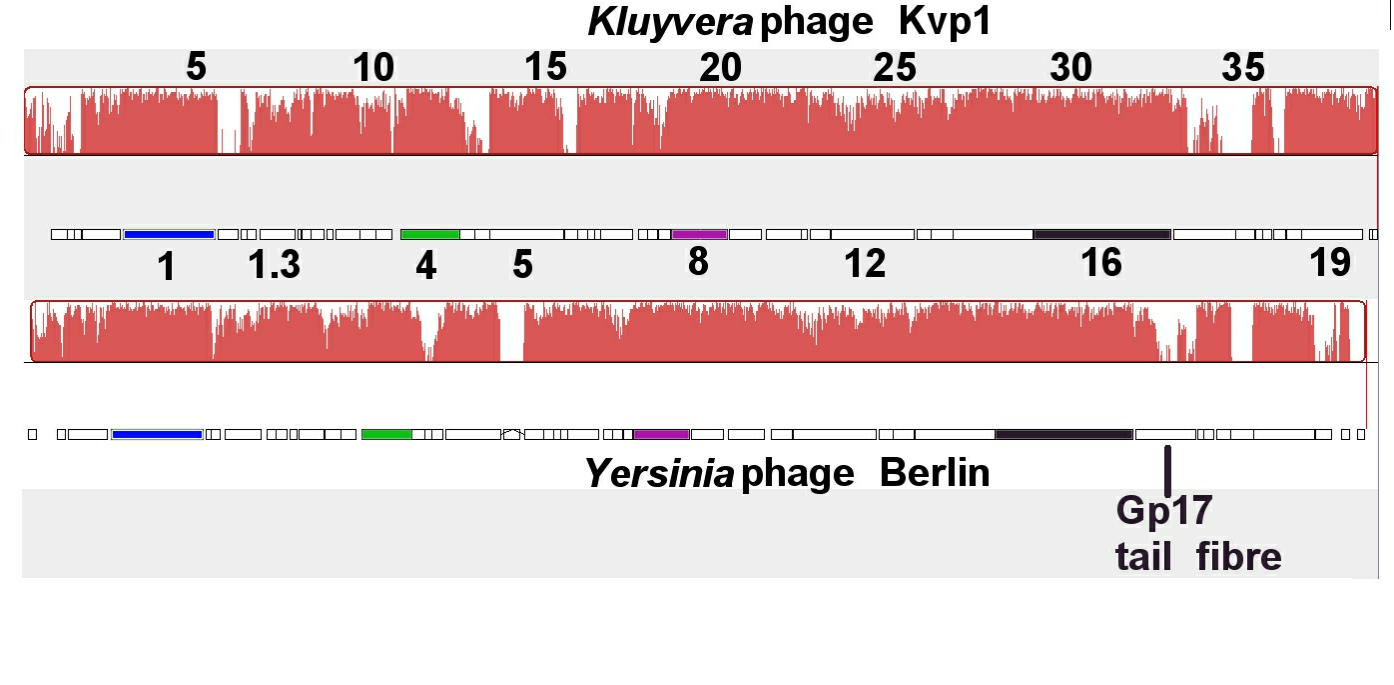
**Comparison of the genomes of *Yersinia *phage Berlin and *Kluyvera *phage Kvp1 using Mauve.** Underneath the name (*Kluyvera *phage Kvp1) is ruler in kb, the degree of sequence similarity, indicated by the intensity of the red region, and, the gene map with the position of 8 genes indicated.

Several regions of dissimilarity (indicates by areas of white in Figure 1) centred at genes 1.05, 4.7–2.8, 5.3–5.5, 17–17.2, 18.2 and at the left end of the genome are noted. Several of these genes are not found in phage Berlin. The most interesting difference is in gene 17 which encodes the tail fibre. As with other Gp17 homologs sequence similarity is only found at the N-terminus, the part of the protein which is associated with the tail structure. The C-terminus is involved in ligand interactions and exhibits considerable differences.

**Figure 2:**
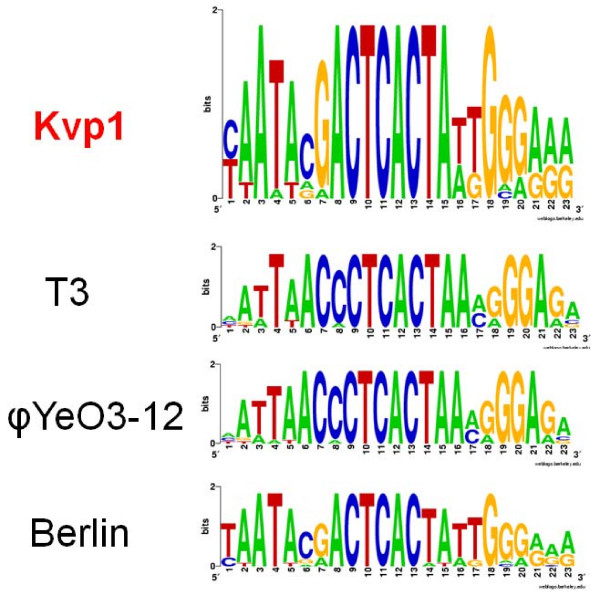
**Weblogos**[**27**]**of some T7-like phage-specific promoters created online at ****showing that the Kvp1 promoters are most closely related to those of phage Berlin.**

Using CoreGenes Kvp1 shares 37 (61.7%), 12 (23.1%) and 9 (18.4%) homologs with the type phages of the three *Autographivirinae *genera – T7, Sp6 and φKMV-like viruses. The results indicate that Kvp1 is a member of the T7-like virus genus. A comparison with the proteome of phage Berlin indicates 37 homologs – 82.2% common proteins. While the percentage of common proteins is less when compared with coliphage T3 (70.9% similarity) the early regions of T3 and Kvp1 are very similar in that Kvp1 encodes T3-like Gp0.3, 0.6 and 0.7 homologs. The product of early gene 0.3 (Ocr) is a small protein which mimics B-form DNA and binds to, and inhibits, type I restriction endonucleases [9,10]; and, possesses S-adenosyl-L-methionine hydrolase activity [11]. Gp0.7 produces functions in host gene shutoff [12] and as a protein kinase which phosphorylates host elongation factors G and P and ribosomal protein S6 [13]. A major dissimilarity between Kvp1 and T3/T7 is that while the latter phages possess multiple strong promoters recognized by the host RNA polymerase, only a single promoter showing homology to the consensus was found in the Kvp1 genome. In keeping with the protein similarity to *Yersinia *phage Berlin, the phage specific promoters are also most closely related in sequence to those of this bacterial virus (Fig. 2).

**Figure 3:**
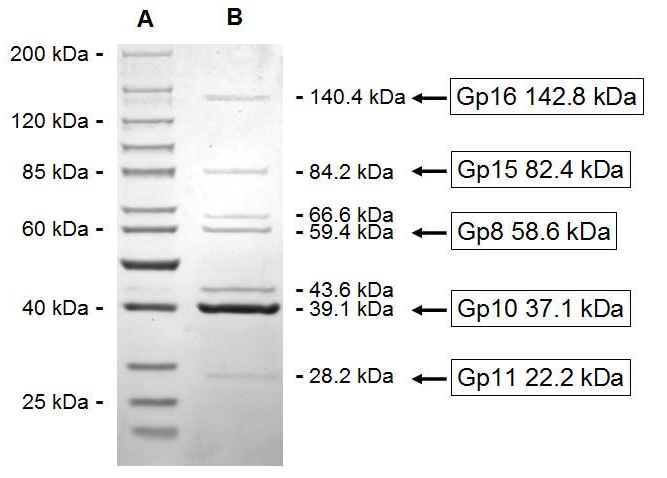
**Denaturing SDS-PAGE of bacteriophage Kvp1 structural proteins (LaneB) alongside the protein marker (Lane A).** The masses of the proteins are indicated in the adjacent lanes. The tentative identification based on *in silico *analysis of the properties of the gene products (Gp) are indicated on the right.

Typical of this type of bacteriophage, Kvp1 displays a simple protein profile (Fig. 3) in which most of the protein bands can be assigned based upon the extensive knowledge of these phages, and the mass of the protein bands compared with the in silico analysis of the proteins based upon genomic analysis.

**Figure 4:**
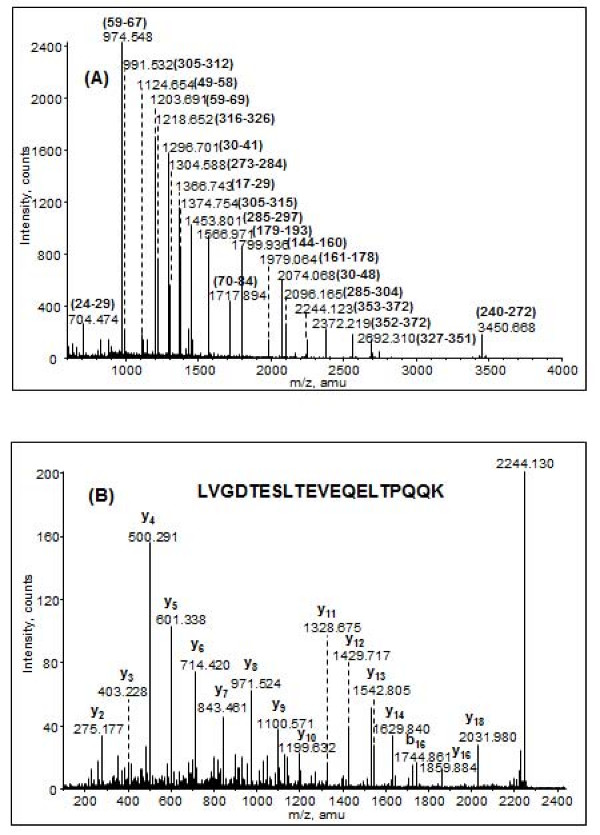
**MALDI QqTOF MS and MS/MS analyses on the in-gel tryptic digest of the 43.6 kDa protein band.** (A) MS spectrum of the trypsin digested peptides. The corresponding Gp10B peptides are shown in the parenthesis. (B) MS/MS sequencing of a typical peptide at m/z 2244.13 yielded a series of C-terminal y fragments (labelled on the top of the fragment ions) which identified the peptide sequence containing the residues 353–372.

One band of interest, with a mass of 43.6 kDa, was noted migrating just above that of the major capsid protein (Gp10) which was not immediately linked, on the basis of its mass, to a product of one of the morphogenesis genes. One of the characteristics of some T7-like phages is that they display, on SDS-PAGE, two "versions" of the major capsid protein which are designated as 10A and 10B [14]. The sequences of the amino termini of these proteins are identical, but during translation a rare ribosomal slippage occurs permitting the elongation of the protein product. The features of this system are a protein slightly larger than the capsid, a slippery site in the DNA/RNA and a downstream stem-loop structure capable of forming a pseudoknot [15,16]. We obtained evidence for a potential pseudoknot using pknotsRG at [17] located 144 bp downstream from the end of the capsid gene, but no typical slippage site was observed. The nature of this protein was investigated by in-gel enzymatic digestion and high-resolution mass spectrometry. MALDI QqTOF MS analysis on a tryptic digest has yielded 70% sequence coverage of the protein Gp10 (Figure 4), and three unique peptides were present at m/z 2244.123, m/z 2372.219 and m/z 2692.310 which revealed the distinct C-terminal amino acid residues 327–372 from the protein sequences of Gp10. This indicates that Kvp1 produces a major capsid protein (10A) and a minor protein (10B) through programmed -1 frameshifting at TTTTCA. The Gp10B protein is predicted to have a calculated mass of 42.1 kDa, consistent with the estimated value of 43.6 kDa by SDS-PAGE gel.

## Conclusion

Our data conclusively demonstrate that *Kluyvera *virus Kvp1 is a member of T7-like virus genus of the *Podoviridae *subfamily *Autographivirinae*. It differs from phages such as T3 and φYeO3-12 which exhibit capsid frameshifting at lysyl residues, by ribosomal slippage at polyU residues (phenylalanine) – a property it shares with *Yersinia *phage φA1122.

## Materials and methods

### Purification of phage wV8

Bacteriophage Kvp1 (HER400) and its host *K. cryocrescens *strain HER1400 were received from the Felix d'Hérelle Reference Center for Bacterial Viruses at Université Laval (Québec, QC, Canada). The phage was propagated at 30°C using standard protocols, precipitated using polyethylene glycol 8000 and purified through two rounds of CsCl equilibrium gradient centrifugation [18].

### DNA sequencing

The DNA was isolated using the SDS-proteinase K protocol of Sambrook and Russell (2001) and was submitted to the McGill University and Génome Québec Innovation Centre (Montréal, QC, Canada) for DNA sequencing. This resulted in two contigs which were closed using PCR with custom primers and, standard dideoxy sequencing of the amplicon (University of Guelph, Laboratory Services, Guelph, ON, Canada). The termini were determined by primer walking.

### Genome annotation

The genome was screened for tRNA-encoding genes using Aragorn [19] and tRNAScan [20]; and, for protein encoding genes using Kodon (Applied Maths, Austin, TX) and PSI-BLAST [21]. Rho-independent terminators identified using TransTerm [22] at . Phage-specific promoters were discovered using PHIRE [23] and displayed using WebLogo [27]. The sequence of this bacteriophage has been deposited with GenBank (accession no. FJ194439).

### Whole genome comparisons

These were carried out using Mauve [24], and CoreGenes [25].

### Proteomics

SDS-PAGE [26] was carried out on CsCl-purified phage particles along with the PageRuler Unstained Protein Ladder (Fermentas, Burlington, ON, Canada) stained with Coomassie brilliant blue R250 and characterized using Bionumerics software (Applied Maths). Bands were further characterized by in situ trypsin digestion and mass spectrometry. Briefly, the excised gel bands were destained until colorless, and dried using a SpeedVac. Following reduction with DTT and alkylation with iodoacetamide, the protein was digested with 10 ng of sequencing grade trypsin (Calbiochem) in 25 mM NH_4_HCO_3 _(pH 7.6) at 37°C overnight. The proteolytic peptides were extracted, and cleaned up by a C18 Ziptip (Millipore). MALDI data were acquired using an Applied Biosystems/MDS Sciex QStar XL quadrupole time-of-flight (QqTOF) mass spectrometer under a nitrogen laser (337 nm), and 2,5-dihydroxybenzoic acid was used as the matrix. All peptide fingerprinting masses (m/z) on the MS spectrum were compared with the theoretical values generated in-silico by MS-Digest, a ProteinProspector program developed in the UCSF Mass Spectrometry Facility . The individual peptide sequence was identified by MALDI MS/MS measurements on the same instrument using argon as the collision gas.

## Abbreviations

MALDI: matrix-assisted laser desorption ionization; QqTOF MS: quadrupole time-of-flight mass spectrometry; MS/MS: tandem mass spectrometry.

## Competing interests

The authors declare that they have no competing interests.

## Authors' contributions

AMK planned the experiments and prepared the manuscript, EJL propagated and purified the phage; and together with YS contributed to the proteomics. P-JC sequenced the ends of the genome; and AV contributed to the genome annotation.
